# Comparison of optical measurements of critical closing pressure acquired before and during induced ventricular arrhythmia in adults

**DOI:** 10.1117/1.NPh.9.3.035004

**Published:** 2022-08-25

**Authors:** Alec Lafontant, Elizabeth Mahanna Gabrielli, Karla Bergonzi, Rodrigo M. Forti, Tiffany S. Ko, Ronak M. Shah, Jeffrey S. Arkles, Daniel J. Licht, Arjun G. Yodh, W. Andrew Kofke, Brian R. White, Wesley B. Baker

**Affiliations:** a Children’s Hospital of Philadelphia and Perelman School of Medicine at the University of Pennsylvania, Department of Pediatrics, Division of Neurology, Philadelphia, Pennsylvania, United States; b University of Miami Miller School of Medicine, Department of Anesthesiology, Perioperative Medicine and Pain Management, Miami, Florida, United States; c University of Pennsylvania, Department of Physics and Astronomy, Philadelphia, Pennsylvania, United States; d Children’s Hospital of Philadelphia, Department of Anesthesiology and Critical Care Medicine, Philadelphia, Pennsylvania, United States; e Perelman School of Medicine at the University of Pennsylvania, Department of Anesthesiology and Critical Care, Philadelphia, Pennsylvania, United States; f Perelman School of Medicine at the University of Pennsylvania, Department of Medicine, Division of Cardiovascular Medicine, Philadelphia, Pennsylvania, United States; g Children’s Hospital of Philadelphia and Perelman School of Medicine at the University of Pennsylvania, Department of Pediatrics, Division of Pediatric Cardiology, Philadelphia, Pennsylvania, United States

**Keywords:** diffuse correlation spectroscopy, critical closing pressure, intracranial pressure, cerebral blood flow pulsatility

## Abstract

**Significance:**

The critical closing pressure (CrCP) of cerebral circulation, as measured by diffuse correlation spectroscopy (DCS), is a promising biomarker of intracranial hypertension. However, CrCP techniques using DCS have not been assessed in gold standard experiments.

**Aim:**

CrCP is typically calculated by examining the variation of cerebral blood flow (CBF) during the cardiac cycle (with normal sinus rhythm). We compare this typical CrCP measurement with a gold standard obtained during the drops in arterial blood pressure (ABP) caused by rapid ventricular pacing (RVP) in patients undergoing invasive electrophysiologic procedures.

**Approach:**

Adults receiving electrophysiology procedures with planned ablation were enrolled for DCS CBF monitoring. CrCP was calculated from CBF and ABP data by three methods: (1) linear extrapolation of data during RVP (${\mathrm{CrCP}}_{\mathrm{RVP}}$; the gold standard); (2) linear extrapolation of data during regular heartbeats (${\mathrm{CrCP}}_{\text{Linear}}$); and (3) fundamental harmonic Fourier filtering of data during regular heartbeats (${\mathrm{CrCP}}_{\text{Fourier}}$).

**Results:**

CBF monitoring was performed prior to and during 55 episodes of RVP in five adults. ${\mathrm{CrCP}}_{\mathrm{RVP}}$ and ${\mathrm{CrCP}}_{\text{Fourier}}$ demonstrated agreement (R=0.66, slope=1.05 (95%CI, 0.72 to 1.38). Agreement between ${\mathrm{CrCP}}_{\mathrm{RVP}}$ and ${\mathrm{CrCP}}_{\text{Linear}}$ was worse; ${\mathrm{CrCP}}_{\text{Linear}}$ was 8.2±5.9  mmHg higher than ${\mathrm{CrCP}}_{\mathrm{RVP}}$ (mean ± SD; p<0.001).

**Conclusions:**

Our results suggest that DCS-measured CrCP can be accurately acquired during normal sinus rhythm.

## 1 Introduction

Acute brain injury is a leading cause of death and disability in children and adults.^1^^–^^3^ Intracranial hypertension often follows acute injury and is a major cause of secondary brain injury.^4^^–^^7^ The gold standard for its detection is invasive monitoring of intracranial pressure (ICP).^4^^,^^8^^,^^9^ Invasive ICP monitors, however, carry risks of intracranial hemorrhage and infection, and they are not always readily available.^9^^,^^10^ Because invasive cerebral monitoring is restricted to critically ill patients and excludes patients on anticoagulation, non-invasive measurement of ICP could extend improved diagnosis and prognosis to a broader population. Numerous techniques have been proposed to this end; they are broadly categorized as electrophysiologic (e.g., electroencephalogram power spectrum analysis), ophthalmic (e.g., optical coherence tomography of the optic nerve), otic (e.g., otoacoustic emissions), and fluid dynamic [e.g., analysis of transcranial Dopper, diffuse correlation spectroscopy (DCS), and near-infrared (NIR) spectroscopy perfusion signals].^11^^–^^19^ Some of these techniques use non-invasive critical closing pressure (CrCP) measurements of the cerebral circulation as a biomarker of intracranial hypertension and ICP.^14^^,^^18^^–^^23^

CrCP is the isotropic pressure compressing the cerebral arterioles;^23^^,^^24^ it depends on both ICP and vasomotor tone. To date, most studies of CrCP employ transcranial Doppler ultrasound (TCD) to determine cardiac blood velocity waveforms in major arteries such as the middle cerebral artery.^14^^,^^24^^,^^25^ The optical technique of DCS was recently demonstrated as an alternative method for estimating CrCP based on the measurement of arteriolar cerebral blood flow (CBF) through the cardiac cycle.^19^^,^^23^ Importantly, DCS is well-suited for prolonged monitoring at the bedside.^26^^,^^27^

The difference of mean arterial pressure and CrCP is the cerebral perfusion pressure that drives CBF.^24^^,^^28^ Thus, a gold standard procedure for measurement of CrCP would rapidly decrease arterial blood pressure (ABP) to levels for which CBF is zero. This approach is not possible for routine clinical use, but such a scenario does occur during rapid ventricular pacing (RVP) of patients during electrophysiology studies wherein an induced ventricular arrhythmia is performed to identify cardiac tissue causing heart rhythm abnormalities; in this case, CBF approaches zero. This gold standard method of determining CrCP is similar to the method used in a prior TCD study to validate their CrCP measurements.^29^ Herein, using patients undergoing electrophysiology studies, we compared DCS CrCP measurements acquired during normal sinus rhythm (the typical method) against measurements acquired during RVP (the gold standard). During sinus rhythm, CrCP was obtained from (a) linear extrapolation of CBF and ABP data and (b) fundamental-frequency harmonic Fourier filtering of CBF and ABP data. Based on the previous TCD study,^29^ we hypothesize that the CrCP measures obtained during regular heartbeats would correlate with CrCP obtained during RVP and that the use of Fourier filtering would improve the agreement.

## 2 Methods

### 2.1 Experimental Procedures

Seven adult patients undergoing electrophysiology procedures with planned cardiac ablation were enrolled in our study. All subjects provided written consent, and all protocols/procedures were approved by the institutional review board of the University of Pennsylvania, which adheres to the guidelines of the Common Rule and the Food and Drug Administration’s Institutional Review Board and human subject regulations. Electrophysiology studies are performed on patients with ventricular arrhythmia to minimally invasively identify and eliminate tissue in the heart that is causing abnormalities in heart rhythm. During the procedure, at least one catheter is placed into the heart; as part of the procedure, small electric impulses are used to probe the heart wall for abnormalities. During ventricular arrhythmia caused by RVP, cardiac output falls, and ABP approaches CrCP.

**Fig. 1:**
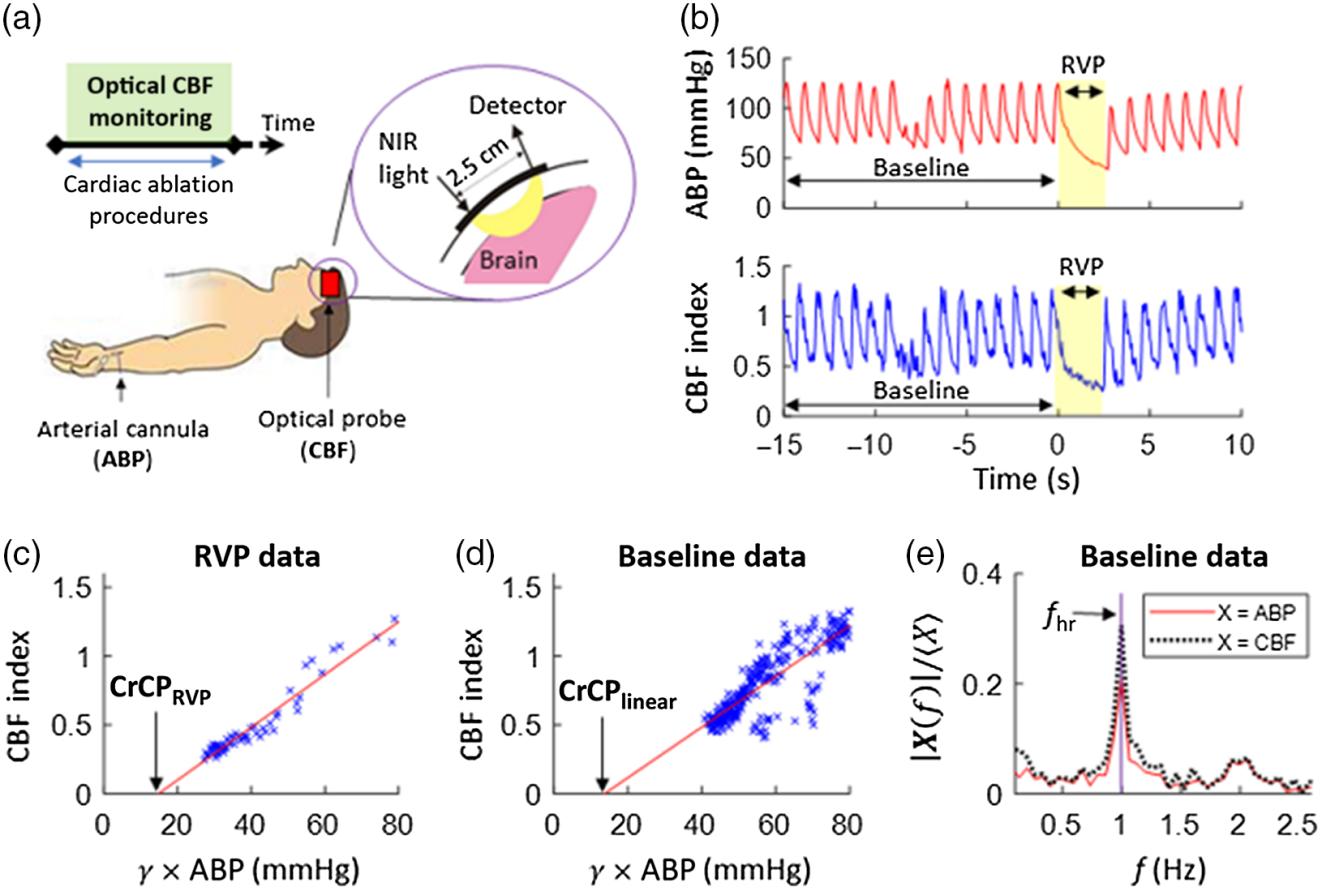
(a) Continuous optical monitoring of CBF was performed with NIR DCS in adult patients undergoing cardiac ablation procedures. During the procedures, arrhythmia is triggered by RVP. (b) Exemplar CBF and ABP time-series data before/during/after an RVP event. For each event, cerebral CrCP was estimated based on CBF and ABP data during baseline and during RVP. (c) CBF plotted against γ ABP during the exemplar RVP episode [shaded in yellow in panel (b)]. γ ABP is the in-flow blood pressure at the entrance to the arteriole compartment (γ=0.6 is assumed; see main text). The solid red line is the linear best fit, and its x-intercept provides an estimate of CrCP, i.e., ${\mathrm{CrCP}}_{\mathrm{RVP}}$ (the gold standard). (d) CBF plotted against γ ABP during the 15-s baseline interval prior to the exemplar RVP episode. The x-intercept of the linear best-fit line provides another estimate of CrCP, i.e., ${\mathrm{CrCP}}_{\text{Linear}}$. (e) Fourier spectral amplitudes of the baseline ABP and CBF data, normalized by their respective means, plotted against frequency (f). A third estimate of CrCP, i.e., ${\mathrm{CrCP}}_{\text{Fourier}}$ (see Eq. (2)), is derived using these normalized amplitudes at the heart rate ($f_{\mathrm{hr}}$). The unit of the DCS-derived CBF index is $10^{-8}\;{\mathrm{cm}}^{2}/s$.

At the time of the electrophysiology study, after induction of anesthesia, a DCS optical probe was placed on the forehead to monitor CBF in brain supplied by the anterior middle cerebral artery for the entire cardiac ablation procedure [Fig. 1(a)]. CBF was measured continuously at 20 Hz using a custom-built DCS instrument with a software correlator that is described elsewhere.^30^^,^^31^ Specifically, DCS intensity autocorrelation measurements at 2.5 cm source-detector separation and 785 nm wavelength were averaged across four detection channels. Using standard techniques,^32^ CBF was obtained from a semi-infinite fit to the autocorrelation measurement using an assumed tissue optical absorption coefficient of $0.1\;{\mathrm{cm}}^{-1}$ and reduced scattering coefficient of $8\;{\mathrm{cm}}^{-1}$. Fractional blood flow changes obtained with DCS, which are used to calculate CrCP (Sec. 2.2), are robust to errors in these assumed tissue optical properties.^33^

ABP was simultaneously measured by a radial artery catheter attached to a FloTrac sensor (Edwards Lifesciences). The FloTrac sensor provides two separate analog outputs of the same ABP signal. One output was connected to the standard vital signs monitor used by the clinical team, while the other output was connected to an analog input of our DCS instrument’s DAQ board (PCIe6323, National Instruments). The ABP and CBF measurements were synchronized and recorded at 20 Hz sampling.

### 2.2 Data Processing

The ABP waveform measurement temporally lagged the CBF waveform measurement because of the differences in distance between the arm arterial tree (where the ABP measurements were made) and heart versus cerebral arterial tree and heart; i.e., it takes longer for ABP cardiac waveforms to reach the arm than the brain. For each patient, we used a cross-correlation technique to measure this lag and align the ABP and CBF time series (xcorr, MATLAB R2018a, Mathworks). The key data of interest for computing CrCP are the continuous high-time-resolution CBF and ABP waveform data (20 Hz sampling) before and during episodes of RVP. We only considered episodes of RVP that resulted in a gap between typical systolic ABP waves of greater than 1.5 s. For each included episode, CrCP was computed during the drop in ABP (the gold standard method). This measurement was compared with calculations of CrCP obtained from data during the 15 s of sinus rhythm data just prior to RVP. ABP and CBF data during an exemplary episode are shown in Fig. 1(b).

Our use of a linear resistive model for the cerebral arteriole compartment between the large arteries and capillaries (i.e., the compartment measured by DCS) underlies the computation of CrCP with the gold standard and normal sinus rhythm methods. Specifically, in this model, the in-flow blood pressure at the entrance to the arteriole compartment is γABP (γ=0.6 was assumed^34^), the out-flow blood pressure at the distal end of the arteriole compartment is CrCP, and the arteriolar resistance is r. Then, using an Ohms’ law relation for blood flow, the connection between measured ABP and DCS-CBF at time t is given by^23^

γABP(t)−CrCP=CBF(t)r. (1)

Equation (1) assumes that CrCP and r remain constant over the time-scale of the measured fluctuations in ABP and CBF (i.e., CrCP and r remain constant during RVP and during the 15 s of sinus rhythm data prior to RVP).

For the gold standard method to compute CrCP during RVP (i.e., ${\mathrm{CrCP}}_{\mathrm{RVP}}$), we performed a linear regression of the continuous CBF versus γABP data acquired during the long diastole accompanying RVP [see Fig. 1(c)]; in Fig. 1(c), the continuous synchronized CBF and γABP data during RVP are plotted on the vertical and horizontal axes, respectively. This procedure enabled us to find the extrapolated value of γABP for which CBF is zero; this is the CrCP [Eq. (1)]. We denote CrCP computed this way as ${\mathrm{CrCP}}_{\mathrm{RVP}}$ (i.e., the x-intercept of the linear regression fit). Although ${\mathrm{CrCP}}_{\mathrm{RVP}}$ is extrapolated, we consider it to be the gold standard measurement because CBF approaches close to zero under these conditions.

We used two methods to compute CrCP during the 15 s of regular heartbeat data (normal sinus rhythm) just prior to RVP. In one method, similar to the computation of ${\mathrm{CrCP}}_{\mathrm{RVP}}$, we performed a linear regression of CBF versus γABP to find the (extrapolated) blood pressure at which CBF is zero [Fig. 1(d)]. We denote CrCP computed this way as ${\mathrm{CrCP}}_{\text{Linear}}$. In the second method, we employed fundamental harmonic Fourier filtering of the 15 s of ABP and CBF data to compute CrCP (i.e., denoted as ${\mathrm{CrCP}}_{\text{Fourier}}$). As discussed in detail elsewhere,^23^^,^^24^^,^^29^^,^^35^ in our vascular model, the ratio of the Fourier amplitudes of the fundamental harmonics of the ABP and CBF waveforms multiplied by γ, i.e., $\gamma |\mathrm{ABP}(f_{\mathrm{hr}})|/|\mathrm{CBF}(f_{\mathrm{hr}})|$, is the arteriolar resistance r. ($f_{\mathrm{hr}}$ is the heart rate frequency.) Substituting this into Eq. (1), we obtain

$${\mathrm{CrCP}}_{\text{Fourier}}=\gamma \langle \mathrm{ABP}\rangle (1-\frac{\left|\mathrm{ABP}(f_{\mathrm{hr}})|/\langle \mathrm{ABP}\right\rangle }{\left|\mathrm{CBF}(f_{\mathrm{hr}})|/\langle \mathrm{CBF}\right\rangle }),$$ (2)

where the angular brackets, ⟨ ⟩, represent the means across the 15 s of heartbeat data. Exemplary measurements of $\left|\mathrm{ABP}(f_{\mathrm{hr}})|/\langle \mathrm{ABP}\right\rangle$ and $\left|\mathrm{CBF}(f_{\mathrm{hr}})|/\langle \mathrm{CBF}\right\rangle$ are shown in Fig. 1(e). The Fourier amplitudes were obtained from the discrete Fourier transforms of the time-series data (fft, MATLAB R2018a).

### 2.3 Statistical Analysis

Summary statistics are presented using means and standard deviations for ${\mathrm{CrCP}}_{\mathrm{RVP}}$, ${\mathrm{CrCP}}_{\text{Linear}}$, ${\mathrm{CrCP}}_{\text{Fourier}}$, mean ABP prior to RVP, DCS detected photon count rate prior to RVP, and the temporal lag of the ABP waveform relative to the CBF waveform. We carried out both linear regression and Bland–Altman analyses to assess agreement between (a) ${\mathrm{CrCP}}_{\text{Linear}}$ and ${\mathrm{CrCP}}_{\mathrm{RVP}}$ and (b) ${\mathrm{CrCP}}_{\text{Fourier}}$ and ${\mathrm{CrCP}}_{\mathrm{RVP}}$. We further used paired t-tests to assess whether ${\mathrm{CrCP}}_{\text{Linear}}$ differed from ${\mathrm{CrCP}}_{\mathrm{RVP}}$ and whether ${\mathrm{CrCP}}_{\text{Fourier}}$ differed from ${\mathrm{CrCP}}_{\mathrm{RVP}}$. For all statistical tests, a p value of <0.05 was deemed to represent statistical significance. All calculations were carried out using MATLAB R2018a.

The use of Fourier filtering requires data spanning multiple cardiac cycles. Therefore, when longer time windows are used to compute the Fourier amplitudes, the frequency resolution for the heart rate is higher. As a check to assess the sensitivity ${\mathrm{CrCP}}_{\text{Fourier}}$ to the 15 s window length, we computed ${\mathrm{CrCP}}_{\text{Fourier}}$, as described earlier, using different window lengths prior to RVP (i.e., 10, 15, 30, 60, 120, and 180 s). We examined agreement between the computations of ${\mathrm{CrCP}}_{\text{Fourier}}$ with different window lengths using an intraclass correlation coefficient (ICC). ICC was computed based on a single-measurement, absolute-agreement, two-way mixed-effects model.^36^ In addition, we were interested in whether there was better agreement between the computations of ${\mathrm{CrCP}}_{\text{Fourier}}$ with subsets of longer window lengths. To this end, we used ICCs to assess agreement between the computations of ${\mathrm{CrCP}}_{\text{Fourier}}$ using the following subsets of longer window lengths: (a) 15, 30, 60, 120, and 180 s and (b) 30, 60, 120, and 180 s.

In another secondary analysis, we used a Pearson correlation coefficient (R) to quantify the strength of the linear correlation between ${\mathrm{CrCP}}_{\text{Linear}}$ and ${\mathrm{CrCP}}_{\text{Fourier}}$, and we used a paired t-test to assess whether ${\mathrm{CrCP}}_{\text{Linear}}$ differed from ${\mathrm{CrCP}}_{\text{Fourier}}$.

In our final secondary analysis, we quantified the strength of the linear correlations between the CBF and ABP time series during RVP and during the 15 s prior to RVP. Strong linear correlations support the validity of our assumption of constant arteriolar resistance for computing CrCP [Eq. (1)]. For each time-period, we computed the mean (95% CI) of R across all RVP episodes. Because Pearson correlation coefficients are not normally distributed and are bound to be between −1 and 1, we first calculated the mean and standard deviation of the mean after transformation of the coefficients using Fisher transforms (i.e., ⟨F⟩ and $F_{\mathrm{SD}}/\sqrt{n}$, where F=arctanh(R) is the Fisher-transformed value, n is the number of RVP episodes, and arctanh is the hyperbolic arctangent).^37^^,^^38^ The resulting averaged values for each time period were then transformed back to the correlation space with the hyperbolic tangent (tanh). Specifically, the Pearson correlation mean (95% CI) is $\tanh(\langle F\rangle )\;(\tanh(\langle F\rangle -1.96F_{\mathrm{SD}}/\sqrt{n}),\tanh(\langle F\rangle +1.96F_{\mathrm{SD}}/\sqrt{n}))$.

## 3 Results

Seven patients were enrolled (four males and three females), and their average age was 65±4 years. Fifty-five episodes of RVP across five patients were included. The RVP episodes in the other two enrolled patients were all less than 1.5 s and thus were excluded. Across the 55 episodes, the average mean ABP and DCS detected photon count rate prior to RVP were 85±21  mmHg and 62±28  kHz, respectively. The ABP waveform lagged the CBF waveform by 0.22±0.14  s.

**Fig. 2:**
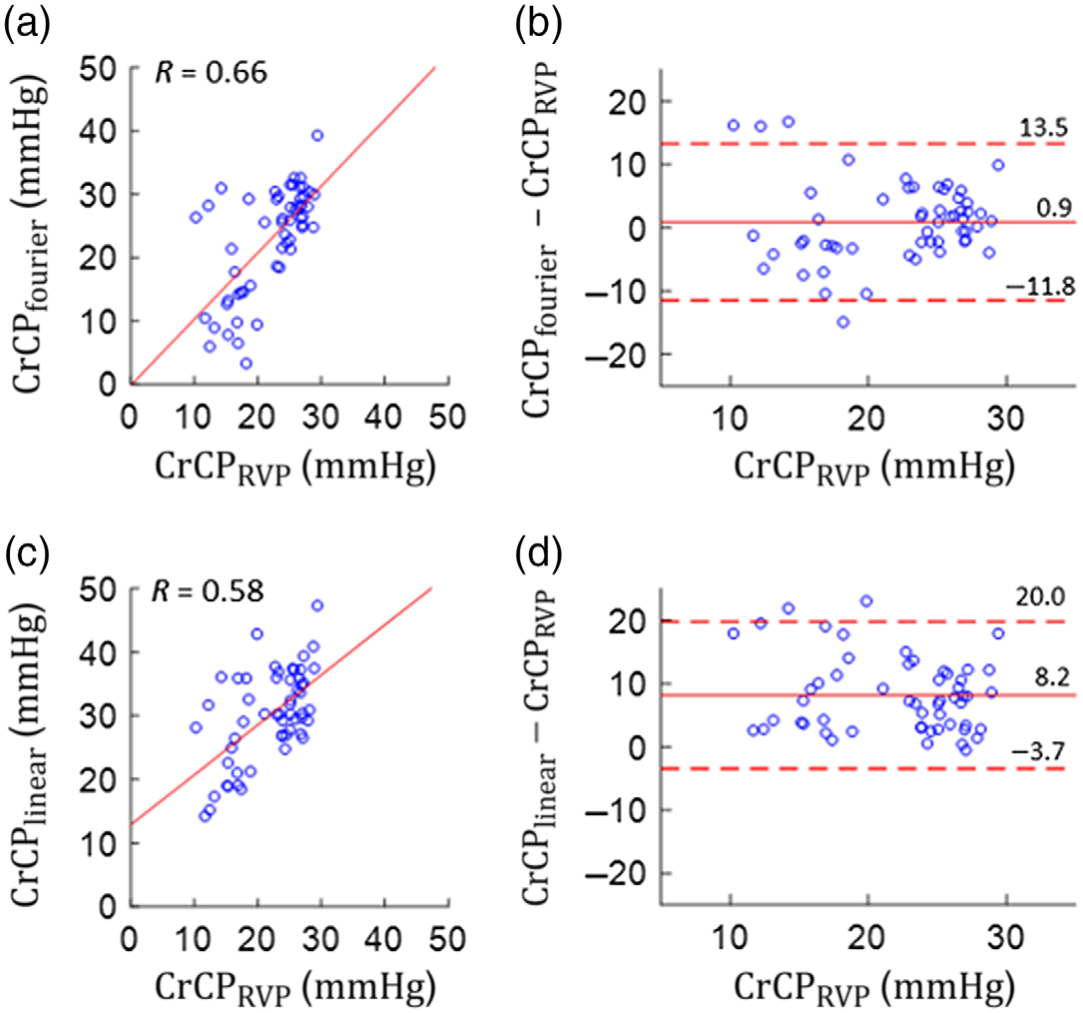
CrCP was derived based on CBF and ABP data during RVP, i.e., ${\mathrm{CrCP}}_{\mathrm{RVP}}$, which we consider to be the gold standard. CrCP was also derived from CBF and ABP data during the 15-s of regular heart beats prior to RVP via Fourier filtering (i.e., ${\mathrm{CrCP}}_{\text{Fourier}}$) and linear extrapolation (i.e., ${\mathrm{CrCP}}_{\text{Linear}}$). Measurements were made for n=55 RVP episodes across five adults. (a) ${\mathrm{CrCP}}_{\text{Fourier}}$ plotted against ${\mathrm{CrCP}}_{\mathrm{RVP}}$ with the linear best-fit line. (b) Bland-Altman plot of the difference between ${\mathrm{CrCP}}_{\text{Fourier}}$ and ${\mathrm{CrCP}}_{\mathrm{RVP}}$. (c) ${\mathrm{CrCP}}_{\text{Linear}}$ plotted against ${\mathrm{CrCP}}_{\mathrm{RVP}}$ with the linear best-fit line. (d) Bland-Altman plot of the difference between ${\mathrm{CrCP}}_{\text{Linear}}$ and ${\mathrm{CrCP}}_{\mathrm{RVP}}$. The Bland-Altman plots show the mean difference (solid horizontal line) ± 1.96 standard deviations of the difference (dashed horizontal lines).

The average ${\mathrm{CrCP}}_{\mathrm{RVP}}$, ${\mathrm{CrCP}}_{\text{Linear}}$, and ${\mathrm{CrCP}}_{\text{Fourier}}$ measurements across the included RVP episodes were 22.0±5.3, 30.2±7.2, and 22.9±8.4  mmHg, respectively. A linear regression and Bland–Altman analysis showed agreement between ${\mathrm{CrCP}}_{\text{Fourier}}$ and ${\mathrm{CrCP}}_{\mathrm{RVP}}$ [Figs. 2(a) and 2(b), Table 1]. The two parameters were significantly correlated (R=0.66, p<0.001), the slope and intercept of the linear regression were not significantly different from unity and zero, respectively, and their mean difference was not significantly different from zero. Agreement was worse between ${\mathrm{CrCP}}_{\text{Linear}}$ and ${\mathrm{CrCP}}_{\mathrm{RVP}}$ [Figs. 2(c) and 2(d) and Table 1]. Although the two parameters were significantly correlated (R=0.58, p<0.001), ${\mathrm{CrCP}}_{\text{Linear}}$ overestimated ${\mathrm{CrCP}}_{\mathrm{RVP}}$ by 8.2±5.9  mmHg (p<0.001).

**Table 1:** Linear relationships between the normal sinus rhythm CrCP (i.e., ${\mathrm{CrCP}}_{\text{Fourier}}$ or ${\mathrm{CrCP}}_{\text{Linear}}$) and gold standard CrCP (i.e., ${\mathrm{CrCP}}_{\mathrm{RVP}}$) measurements and the normal sinus rhythm minus gold standard CrCP difference.

Parameter	Slope (95% CI)	Intercept (mmHg) (95% CI)	Pearson R, p-value	Difference ( mmHg ) (Mean ± SD), p-value
${\mathrm{CrCP}}_{\text{Fourier}}$	1.05 (0.72, 1.38)	−0.2 (−7.6, 7.2)	0.66, p<0.001	0.9 ± 6.3, p=0.3
${\mathrm{CrCP}}_{\text{Linear}}$	0.79 (0.49, 1.09)	12.9 (6.1, 19.7)	0.58, p<0.001	8.2 ± 5.9, p<0.001

In a secondary analysis, we varied the window length used to compute ${\mathrm{CrCP}}_{\text{Fourier}}$ to values different by 15 s (i.e., 10, 30, 60, 120, and 180 s). The ICC between the six sets of ${\mathrm{CrCP}}_{\text{Fourier}}$ data (i.e., one data set for each window length) was 0.43 (95% CI, 0.23 to 0.61). This poor overall agreement in ${\mathrm{CrCP}}_{\text{Fourier}}$ computed using these different time windows is driven largely by data from one window length, i.e., the 10-s window. The ICC between the five sets of ${\mathrm{CrCP}}_{\text{Fourier}}$ data computed with the longer windows (i.e., 15, 30, 60, 120, and 180 s) was substantially higher, i.e., 0.76 (95% CI, 0.68 to 0.84). This higher ICC was, in turn, not different from the ICC between the four sets of ${\mathrm{CrCP}}_{\text{Fourier}}$ data computed with even longer windows (i.e., 30, 60, 120, and 180 s), i.e., 0.85 (95% CI, 0.81 to 0.89). Together, these results suggest that a 15-s window is sufficiently long to compute ${\mathrm{CrCP}}_{\text{Fourier}}$ in adults and that windows of 10-s or less are too short.

In another secondary analysis, a significant linear correlation of R=0.61 between ${\mathrm{CrCP}}_{\text{Linear}}$ and ${\mathrm{CrCP}}_{\text{Fourier}}$ was observed (p<0.001), and the ${\mathrm{CrCP}}_{\text{Linear}}-{\mathrm{CrCP}}_{\text{Fourier}}$ difference of 7.3±6.9  mmHg (mean ± SD) was greater than zero (p<0.001).

In our third secondary analysis, significant linear correlations between ABP and CBF during RVP and prior to RVP were observed. The mean (95% CI) linear correlation between the ABP and CBF data during RVP was R=0.91 (0.89, 0.92). The corresponding mean linear correlation between the ABP and CBF data acquired during the 15-s interval prior to RVP was R=0.82 (0.78, 0.85). The strong linear correlations support our assumption of constant arteriolar resistance for computing CrCP [see Eq. (1)].

## 4 Discussion

The optical method for deriving CrCP relies on measurements of CBF and ABP variations over a time scale for which arteriolar resistance is constant. Herein, we compared CrCP estimates obtained from two distinct causes of fast CBF/ABP variations: normal sinus rhythm and RVP. We treated the latter estimate (${\mathrm{CrCP}}_{\mathrm{RVP}}$) as the gold standard because, during RVP, ABP approaches the true CrCP. Importantly, we found that, during the regular heartbeats, CrCP estimated from fundamental harmonic Fourier filtering (i.e., ${\mathrm{CrCP}}_{\text{Fourier}}$) agreed with ${\mathrm{CrCP}}_{\mathrm{RVP}}$. Agreement was worse for CrCP estimates based on linear extrapolation during regular heartbeats (i.e., ${\mathrm{CrCP}}_{\text{Linear}}$).

The advantages of the Fourier method include its insensitivity to errors in temporal alignment between the CBF and ABP waveforms and its removal of higher order harmonics from the waveforms (in contrast to the linear method where all harmonics are present). During normal sinus rhythm, the CBF and ABP waveforms are repetitive periodic oscillations. Accordingly, the waveforms can be represented by a Fourier series with frequencies (or harmonics) that are integral multiples of the frequency of repetition (i.e., the fundamental harmonic). The fundamental harmonic is the heart rate ($f_{\mathrm{hr}}$). The removal of higher harmonics (i.e., $2f_{\mathrm{hr}},3f_{\mathrm{hr}},\ldots$) is advantageous because vascular compliance effects are more pronounced at higher signal frequencies, i.e., the single-resistor vascular model used to calculate CrCP is less accurate for larger harmonics.^19^ In addition, both the DCS and TCD CrCP measurement techniques make the approximation that the ABP waveforms in the arm (where the ABP measurements were made) and cerebral arterial trees are equivalent. In reality, there are subtle differences between the shapes of the ABP waveforms in the arm and cerebral arterial trees. These subtle differences primarily affect the higher harmonics in the ABP waveform.^39^ Thus, fundamental harmonic Fourier filtering renders the CrCP measurement less sensitive to errors that arise from ABP waveform estimation.

These reasons could explain why ${\mathrm{CrCP}}_{\text{Linear}}$ overestimated the gold standard. Accordingly (especially because the gold standard method is not usually available), we recommend use of the fundamental harmonic Fourier filtering method to measure CrCP during routine use. We note that another recently proposed DCS-based method to estimate CrCP has also shown promise. This method, employing an innovative algorithm to resolve pulsatile CBF waveforms at 100 Hz sampling with DCS, used linear extrapolation solely during diastole between regular heartbeats to determine CrCP (i.e., CrCP was the x-intercept of the best-fit line to CBF versus ABP data after ABP drops below the dicrotic notch).^19^ The CBF sampling rate in our study was too low to estimate CrCP in this manner. Future work is needed to assess whether this diastole-only method for calculating CrCP improves agreement with the gold standard CrCP estimate.

Our results indicate that DCS CrCP measurements during normal sinus rhythm are accurate in adults with presumed normal ICP levels. Future work is needed, however, to assess the impact of ICP pulsatility on CrCP measurements in adults with intracranial hypertension. Both the DCS and TCD methods for deriving CrCP assume that CrCP is constant on the time scale of CBF and ABP fluctuations [Eq. (1)]. In practice, $\left|\mathrm{CrCP}(f_{\mathrm{hr}})\right|$ is greater than zero because of the pulsatility in ICP caused by the transfer of systolic ABP increases into brain tissue.^40^ From Eq. (1), the arteriolar resistance is more precisely given as $r=(\gamma |\mathrm{ABP}(f_{\mathrm{hr}})|-|\mathrm{CrC}P(f_{\mathrm{hr}})|)/|\mathrm{CB}F(f_{\mathrm{hr}})|$. The neglect of $\left|\mathrm{CrCP}(f_{\mathrm{hr}})\right|$ results in overestimation of R and underestimation of CrCP. At normal ICP levels, typical ICP pulsatility is small (∼1  mmHg),^40^ but it can become more substantial during intracranial hypertension.^40^ On a related note, ICP pulsatility can also induce subtle oscillations in intracranial tissue volume (i.e., the ICP-volume compliance curve),^40^ which may cause motion artifacts in DCS measurements. The observed agreement between ${\mathrm{CrCP}}_{\text{Fourier}}$ and ${\mathrm{CrCP}}_{\mathrm{RVP}}$ suggests that these artifacts do not significantly impact measurements at normal ICP levels, but further investigations at higher ICP pulsatility levels are warranted.

The DCS CrCP measurement method has notable advantages compared with the TCD method.^19^^,^^23^ DCS is well-suited for prolonged monitoring at the bedside,^26^^,^^27^ less susceptible to the confound of turbulent flow in the vasculature^41^ (the Reynolds number of flow in arterioles is substantially lower than the Reynolds number in large arteries^42^), and more sensitive to localized injuries.^19^^,^^23^ One limitation is the need to assume the γ coefficient in Eq. (1). This accounts for the blood pressure drop across the large cerebral arteries. Our assumed γ of 0.6 is based on systemic and arteriolar blood pressure measurements in rats.^34^ If the same γ is used for every measurement, then the specific γ assumed will not affect the diagnostic accuracy of detecting intracranial hypertension based on CrCP measurements. It would only alter the specific ${\mathrm{CrCP}}_{\text{Fourier}}$ threshold used for detection. (Note that, in the recent study that introduced the diastole-only method to estimate CrCP from DCS measurements, γ=1 was used.^19^) The analysis is more problematic, however, if γ varies substantially between subjects or within subjects at different timepoints. Future work is needed to investigate the relationship between systemic and arteriolar blood pressure waveforms in humans and large animal models.

## 5 Conclusion

The observed agreement between DCS measurements of cerebral CrCP acquired prior to and during RVP suggests that CrCP can be accurately measured with DCS during regular heartbeats. Further, agreement was better for CrCP estimated from fundamental harmonic Fourier filtering than from linear extrapolation. To further investigate the use of CrCP as a biomarker for intracranial hypertension, future work is needed to study the relation between systemic and arteriolar blood pressure waveforms and to assess the impact of ICP pulsatility on the CrCP calculation during intracranial hypertension.

## Code, Data, and Materials Availability

The data and code presented in this study are available on request from the corresponding author.
